# Parameters of lung inflammation in asthmatic as compared to healthy children in a contaminated city

**DOI:** 10.1186/1471-2466-14-111

**Published:** 2014-07-08

**Authors:** Benigno Linares Segovia, Gabriela Cortés Sandoval, Norma Amador Licona, Juan Manuel Guízar Mendoza, Estela Núñez Lemus, Diana Olivia Rocha Amador, Xóchitl Sofía Ramírez Gómez, Rebeca Monroy Torres

**Affiliations:** 1 Department of Medicine and Nutrition, Division of Health Sciences, University of Guanajuato, León, Guanajuato, México; 2 Department of Teaching and Research PEMEX Regional Hospital Salamanca, Salamanca, Guanajuato, México; 3 UMAE HE 1, Instituto Mexicano del Seguro Social, Leon, Guanajuato, México; 4 Universidad De La Salle Bajío, León, Guanajuato, México; 5 Department of Pharmacy, Division of Natural Sciences, University of Guanajuato, Guanajuato, México; 6 Department of Medicine and Nutrition, University of Guanajuato, 20 de Enero #929, Col. Obregón, León, Guanajuato Z.C. 37320, México

**Keywords:** Air pollution, Lung inflammation, Cysteinyl-leukotrienes, Exhaled breath condensate

## Abstract

**Background:**

The impact of air pollution on the respiratory system has been estimated on the basis of respiratory symptoms and lung function. However; few studies have compared lung inflammation in healthy and asthmatics children exposed to high levels of air pollution. The aim of the study was to elucidate the modulatory effect of air pollution on Cysteinyl-leukotrienes (Cys-LTs) levels in exhaled breath condensate (EBC) among healthy and asthmatic children.

**Methods:**

We performed a cross-sectional comparative study. Children between 7–12 years of age, asthmatics and non-asthmatics, residents of a city with high levels of PM_10_ were included. In all cases, forced spirometry, Cys-LTs levels in EBC, and the International Study of Asthma and Allergies in Childhood questionnaire were evaluated. We also obtained average of PM_10_, CO, SO_2_ and O_3_ levels during the period of the study by the State Institute of Ecology.

**Results:**

We studied 103 children (51 asthmatics and 52 non-asthmatics). Cys-LTs levels were higher in asthmatics than in non-asthmatics (77.3 ± 21.6 versus 60.3 ± 26.8 pg/ml; p = 0.0005). Also, Cys-LTs levels in children with intermittent asthma were lower than in children with persistent asthma (60.4 ± 20.4 versus 84.7 ± 19.2 pg/ml; p = 0.0001). In the multiple regression model, factors associated with levels of Cys-LTs were passive smoking (β = 13.1, p 0.04) and to be asthmatic (β = 11.5, p 0.03).

**Conclusions:**

Cys-LTs levels are higher in asthmatic children than in healthy children in a contaminated city and its levels are also associated with passive smoking.

## Background

Air pollution has become a global public health problem. It is considered by the World Health Organization (WHO) as a global health priority, according to a recent study, the air pollution is responsible for 1.4% of all deaths in the world [1].

Epidemiological studies show that exposure to air pollution, is associated with an increased incidence and severity of asthma [2,3], acute respiratory infections [4,5], pulmonary function decline [6] and other chronic obstructive pulmonary disease [7,8].

Most of literature has taken as health indicators, hospital admissions or attendance at the emergency services. However, not all people are exposed to this impact on health in the same conditions; the most vulnerable groups are children and elderly people. The child population has certain characteristics that make them more susceptible to environmental impact unlike adults. Thus, from the point of view of the dose compared with adults, children breathe faster and play outdoors often, therefore, due to its lower weight is greater exposure per unit mass. On the other hand, due to its constant state of development and growth, physiologically immune system and organs are still immature, also irritation and inflammation caused by contaminants easily obstructs their airways [9].

The contaminants frequently associated with asthma exacerbations are: carbon monoxide, ozone, nitrogen dioxide, sulfur dioxide and particulate matter with a diameter of 10 micrometers or less (PM_10_). Continuous exposure to low doses of allergens in patients sensitized causes increased bronchial hyperreactivity, decreased steroid receptor affinity, and therefore, increases the susceptibility to asthma attacks and reduces the response to treatment steroids [3,10].

There are two proposed mechanisms for respiratory disease due to exposure to air pollutants. The first involves a reduction in the forced vital capacity, through to the stimulation of neural receptors in the upper airway due to the release of cyclooxygenase, products of the arachidonic acid [11]. The other mechanism has been linked to the recruitment of inflammatory cells and in general to the inflammation process [12-14].

The impact of air pollution on the respiratory system has been estimated in terms of respiratory symptoms, lung function and other outcomes. However, there are few reports worldwide about the involvement of cysteinyl-leukotrienes (Cys-LTs) in the pathogenesis of lung inflammation [15,16]. The aim of this study was to elucidate the modulatory effect of air pollution on Cysteinyl-leukotrienes (Cys-LTs) levels in exhaled breath condensate (EBC) among healthy and asthmatic children.

## Methods

### Study population

We performed a cross-sectional comparative study. As inflammation biomarker Cisteinyl-leukotrienes in exhaled breath condensate (EBC) was measured. A sample size of 37 subjects per group was calculated (asthmatic and non-asthmatic) using the statistic t, according to detect a difference of at least 10% in the levels of Cys-LTs with an alpha of 0.05, unilateral beta 0.10 and a power of 0.90. Increased 20% for losses (n = 90). We included children 7–12 years of age, asthmatics and non-asthmatics, residents of a city with high levels of PM_10_.

The study took place in the urban area of León, Mexico from September, 2010 to March, 2011. The municipality has a territorial extension of 1 200 km^2^ (3.9% of the total state surface), a population of 1.5 million inhabitants and a population density of 1,250 inhabitants per km^2^. Its climate is temperate most of the time, and it forms part of the industrial corridor of the state. According to reports of the National Institute of Ecology [17], it is one of the cities with high air pollution by PM_10_ (average of 64.5 μg/m^3^ with a range of 41.2-82.6 ug/m^3^). The main producers of this pollutant are sources of area and emissions from motor vehicles.

We studied healthy or asthmatics children who were able to cooperate with the test. Children who had wheeze due to concomitant nonasthmatic chronic airway diseases like cystic fibrosis or patients with asthma who had suffered an exacerbation within a month of the study period were excluded from the study. Children with asthma were recruited from the Pediatric Outpatients Clinics of Regional General Hospital of Leon, Mexico. Normal age-matched control subjects were recruited from a public elementary school located in the same city 2 km away from the monitoring station. None of the participants had been treated with leukotriene inhibitors. Permission was obtained from the authorities of the State Department of Education and from the participating school. The parents, as well as each of the participants, were informed about the objective of the study and the procedures before obtaining consent to participate. The study was approved by the Research Committee of the Department of Medicine and Nutrition of the University of Guanajuato, and was authorized with the registration number 358–10.

### Study design

A detailed history was taken and physical examination performed on each child. Weight and height were recorded to calculate the body mass index (BMI). Asthma was diagnosed when the child had episodic cough, breathlessness, and wheeze responsive to bronchodilators with or without steroids. The medical and sociodemographic history were obtained through the questionnaire proposed by the International Study of Asthma and Allergies in Childhood (ISAAC) validated in a previous study [6].

Lung function was measured by forced spirometry. The spirometry was performed with a spirometer EasyOne® (NDD, Technopark darned Switerland), which meets the diagnostic criteria for precision, accuracy and linearity, established by the American Thoracic Society (ATS, 1994). To carry out follow the recommendations of the ATS and the following parameters were obtained: Forced Vital Capacity (FVC), Forced Expiratory Volume in one second (FEV_1_) and FVC/FEV_1_ ratio. The presence of spirometric values below the fifth percentile was considered abnormal according to height, weight, gender and age.

The quality of spirometric tests was assessed by several criteria in addition to the automatic evaluation done by the software device. One was the number of acceptable maneuvers according to ATS, 1 ranging from 0 to 3, the highest kept by the spirometric software. Another indicator of quality was reproducibility. FEV_1_ and FVC were considered reproducible according to ATS criteria when the best two trials differed by not more than 200 mL. A total of 97.5% of the tests achieved reproducibility within 150 mL fulfilling the 2005 ATS-ERS criteria. Reference values of Hankinson et al. [18] for Mexican-Americans were used, considering that children > 7 years old can fulfill ATS criteria of quality after the first spirometric evaluation. The presence of spirometric values below the 5^th^ percentile of reference values were considered abnormal. The obstructive pattern was defined by the diminution of the FEV_1_ and FEV_1_/FVC index, the restrictive pattern by diminution of the FVC, with normal FEV_1_/FVC index, and mixed pattern by diminution of FVC and FEV_1_.

### Exhaled breath condensate analysis

Exhaled breath condensate collection was performed using the RTube™ EBC collection system (Respiratory Research, Inc, Charlottesville, Virginia, USA). Subjects breathed tidally for 15 minutes, nose clips were not worn, because they are somewhat uncomfortable. The condensate samples were obtained at an average ambient temperature of 13°C. The mean volume collected was 1.2 (range 0.7–1.1.8) ml. The collected condensate was melted and aliquots of 100 μL stored in small plastic tubes at −80°C.

### Biochemical assays

Cys-LTs (LTC4, LTD4, and LTE4) levels were measured with a specific enzyme immunoassay (Cayman Chemical, Ann Arbor, MI), at a wavelength of 410 nm. The lower limit of detection for these assays was 4 pg/ml. The intra-and interassay coefficients of variation of the kits were 10% or less and every sample was assayed by duplicate.

### Data analysis

All data are expressed as means ± SEM or as median and 95% CI according to their normal distribution. Comparison of demographic and clinical data was performed using chi square or Student’s t test according to the type of variable. Comparison of demographic data was done by a chi-square test. To compare the concentration of exhaled Cys considered three groups: non-asthmatics, intermittent asthma and persistent asthma and use analysis of variance with post hoc Tukey test. We used the Pearson correlation test to determine the association between levels of cysteinyl-leukotrienes and spirometric values. Stepwise multiple regression was performed with cysteinyl-leukotriene levels as the dependent variable and group, age, gender, body mass index, the antecedent atopy, exposure to allergens and passive smoking as regressors. Air pollutants were captured as mean values of all the period of the study.

## Results

### Clinical characteristics

One hundred three subjects were enrolled into the study, with a mean age of 9.0 ± 1.3 years. We observed no significant difference in gender distribution between groups. Asthmatics children were older and showed higher BMI than those non-asthmatics. In contrast, they showed lower FEV1 and FEV1/FVC% than healthy children (Table 1).

**Table 1:** Clinical characteristics of the study populations

**Group**	**Asthmatic n = 51**	**No asthmatic n = 52**	**P**
Gender (male/female)	30/22	27/25	0.55
Age (years)	9.5 ± 1.5	8.5 ± 0.9	0.0004
Weight (kg)	36.7 ± 6.0	31.8 ± 6.8	0.004
BMI (kg/m^2^)	18.9 ± 4.2	17.3 ± 2.7	0.02
FVC (liters)	2.2 ± 0.6	2.0 ± 0.3	0.06
FVC (% predicted)	97.8 ± 15.3	98.2 ± 24.1	0.91
FEV1 (liters)	1.7 ± 0.5	1.6 ± 0.2	0.30
FEV1 (% predicted)	86.0 ± 14.7	93.6 ± 10.8	0.003
FEV1/FVC%	78.3 ± 8.3	82.9 ± 9.2	0.01

### Allergic diseases and respiratory symptoms

According to the ISAAC questionnaire, the frequency of respiratory symptoms (cough, wheezing, and rhinorrhea) and allergic (rhinitis and eczema) diseases was significantly higher in asthmatic children (Table 2).

**Table 2:** Prevalence of respiratory symptoms, allergic diseases and lung function disorders

	**Asthmatic n = 51**	**Non-asthmatic n = 52**	**OR**	***p value***
	**No. (%)**	**No. (%)**	**(95% CI)**	
**Respiratory symptoms:**				
Cough	47 (92.1)	13 (25.0)	3.6 (2.2 - 5.9)	0.0001
Wheezing	50 (98.0)	5 ( 9.6)	10.2 (4.4 - 23.4)	0.0001
Rhinorrhea	51 (100)	44 (84.6)	1.1 (1.05 - 1.3)	0.003
**Allergic diseases:**				
Rhinitis	24 (47.0)	3 (5.7)	8.1 (2.6 - 25.4)	0.0001
Eczema	15 (29.4)	1 (1.9)	15.2 (2.1 - 111.5)	0.0001
**Type of impaired lung function:**	16 (31.4)	6 (11.4)	2.7 (1.1 - 6.3)	0.02
Obstructive	10 (19.6)	2 (3.8)	5.2 (1.2 - 22.5)	0.01
Restrictive	1 (1.9)	2 (3.8)	0.65 (0.06 - 6.9)	0.56
Mixed	5 (9.8)	2 (3.8)	2.9 (0.5 - 12.5)	0.22

### Pulmonary function

The spirometry data showed that forced vital capacity in asthmatic children was higher than in non-asthmatics, although this difference was not significant. No significant differences were observed in absolute FEV1. In contrast, the percentage of predicted FEV1 in non-asthmatic children was significantly higher than in asthmatics (p = 0.003). According to spirometric values, lung function abnormalities were higher in the asthmatic group than in non-asthmatics. Also, impaired lung function “obstructive-type”, was more common in asthmatics, but we did not observe significant difference in “restrictive-type” or “mixed-type” disorders between groups (Table 2).

### Air pollutants

According to reports of the State Institute of Ecology, average PM_10_ levels during the period were 196.7 μg/m^3^ with a range of 64.1- 217.9 μg/m^3^ which represents 176 days of measurement. The corresponding averages for other pollutants were: O_3_ = 26.32 μg/m^3^ (15.8-30.9), SO_2_ = 7.8 μg/m^3^ (5.0-11.6), CO = 0.96 ppm (0.57-1.68), and NO_2_ = μg/m^3^ 19.6 (14.6-26.8) in the same period of time.

### Exhaled Cys-LTs

**Figure 1:**
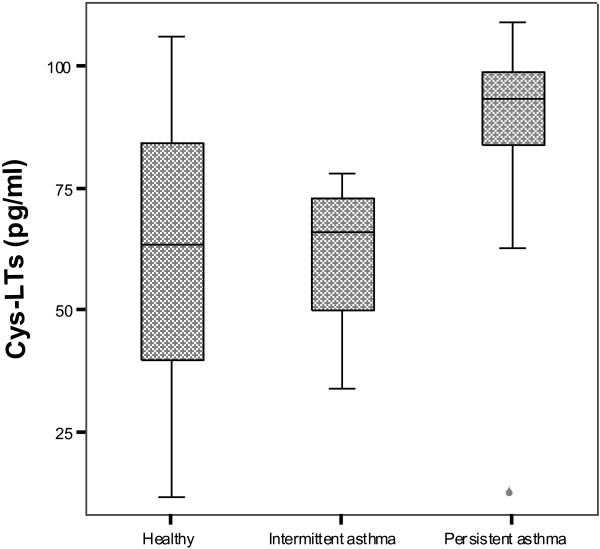
**Cys-LTs levels in exhaled breath in healthy children and those with asthma.** Cys-LTs levels in children with persistent asthma (84.7± 19.2 pg/ml) were significantly higher than in those with intermittent asthma (66.4 ± 20.4 pg/ml; p=0.02) and non-asthmatics (60.4 ± 20.4 pg/ml; p=0.001).

The Cys-LTs levels were detected in the EBC of all children, with mean of 68.7 ± 25.7 pg/ml. The Cys-LTs levels were significantly higher in asthmatics than in non-asthmatics (77.3 ± 21.6 versus 60.3 ± 26.8 pg/ml; p = 0.0005). The average concentration of exhaled Cys-LTs in children with intermittent asthma was significantly lower than in children with persistent asthma (66.4 ± 20.4 versus 84.7 ± 19.2 pg/ml; p = 0.02). The analysis of variance with post hoc Tukey test showed that Cys-LTs levels in children with persistent asthma were significantly higher than in those with intermittent asthma (p = 0.02) and non-asthmatics (p = 0.001) Figure 1.

There was no correlation between Cys-LTs levels and spirometric parameters (FVC, FEV1, FEV1/FVC%). In the multiple regression model, factors associated with levels of Cys-LTs were passive smoking (β = 13.1, p 0.04) and to be asthmatic (β = 11.5, p 0.03).

## Discussion

It is well documented that high levels of many airborne pollutants can adversely affect many systems of the human body. Epidemiological studies have shown that exposure to air pollutants, even at levels below the standard, are associated with an increased incidence and severity of asthma with pulmonary function decline as well as chronic obstructive pulmonary disease exacerbation.

To know the adverse effects of air pollution on human health, time series ecological studies have been used. In our country there are two multicenter studies conducted in 16 cities, which analyze the effects of air pollutants on mortality (EMECAM) and morbidity (EMECAS project). These works show a significant association between air pollutants and health indicators.

In this study, the prevalence of respiratory symptoms (cough, wheezing, and rhinorrhea) and allergic diseases (rhinitis and eczema) were significantly higher in children with asthma than in healthy children, as well as the prevalence of impaired lung function measured by spirometry. These results are consistent with the well known facts that asthmatic children have more respiratory symptoms, lower lung function, and evidence of airway inflammation compared to non-asthmatic children [4-6] which also increase in places with high air pollution.

The Cys-LTs levels in exhaled breath condensate were higher in children with asthma than in non-asthmatics. These findings agree with those reported by Csoma Z et al. [19] and by Baraldi et al. [20], in healthy and asthmatic schoolchildren 4–16 years old. Also Cys-LTs levels were higher in persistent than in intermittent asthma, these results support that Cys-LTs play an important role in the mechanism of lung inflammation. For example it has been described that they participate in the pathophysiology of airway remodelling [21] and in the pathophysiology of asthma [22]. *In vitro* studies have shown that LTD_4_ augments epidermal growth factor-induced human airway smooth muscle proliferation [23] and that LTC_4_ up regulates collagenase expression and synthesis in human lung fibroblasts [24]. Furthermore, animal models have shown that an increase in airway smooth muscle cells observed in allergen-treated Brown Norway rats was reduced by CysLT_1_ receptor antagonism [25]. So, it seems that current asthma treatment mainly based on disease severity classification has to change more focused on the individual patient [26].

As a relevant observation, Cys-LTs levels in EBC were higher in our study than in others [19,20,27]. Even healthy children showed higher levels (60.3 pg/mL) than those reported by Czoma et al. [19] (18.5 pg/mL) and Baraldi et al. [20] (4.3 pg/mL). This could be related to PM_10_ levels recorded in our city, because it has been reported that PM10 trigger a systemic reaction from lungs to bloodstream in mice [28], and its levels were higher than those considered normal in the Mexican’s Official Norm [29]. Also, in the last three years the average levels of PM10, reported by the Air Quality in Europe in cities of similar studies were 28 μg/m^3^ in London, UK and 49.4 μg/m^3^ in Padova, Italy [30]; significantly lower than in our population (196.7 μg/m^3^). So, it supports additional insight into the toxicity of PM10 and could facilitate shedding light on mechanisms underlying the development of urban air pollution related diseases. Other factors that can also explain this difference are genetics, lifestyle, socioeconomic and geographic location. It is unlikely that the variation between Cys-LTs levels may be due to factors related to the sample processing, because it was collected by the same method, and a similar kit was used.

Another factor related to Cys-LTs levels was passive smoking. Supporting this relationship, recently the exposure to environmental tobacco smoke (ETS), as assessed by urinary cotinine levels, was associated with an increased urinary concentration of LTE (4) [31]. Also, it has been reported that ETS modifies the acute effects of low-level ambient PM(2.5) exposure on childhood asthma. This negative interaction, the smaller effect of particulate matter exposure in children exposed to higher ETS, may be related to a nonlinear dose–response relationship between asthma mediators and particulate exposures [32].

One limitation of the study is that it was a cross-sectional study; it would be desirable to measure the levels of Cys-LT at least once in every season to determine their variability and association with levels of pollutants and respiratory health damage. Also, in future studies, independent biomarkers of airway inflammation and/or oxidative stress including exhaled nitric oxide [33] and EBC concentrations of isoprostanes [34,35] and metabolites [36,37] should be measured for a more complete assessment of airway inflammation in children exposed to high PM10 levels. Analysis of breath volatile organic compound profiles with electronic noses [38] would be particularly interesting as this technique has been shown to be reproducible and reliable [39], as well as another new specific non-invasive technique for assessing airway inflammation such as nuclear magnetic resonance-based metabolomics of EBC [37]. Also, pharmacological studies aimed at measuring EBC CysLTs after treatment with leukotriene receptor antagonists in asthmatic children exposed to high PM10 levels or passive smoking are required [40].

However, strength in our study is that we performed a re-power calculation using the difference in standard deviations and means in addition to only 10% difference between groups in Cys-LT levels and the result was 93%.

## Conclusions

Cys-LTs levels are higher in asthmatic children than in healthy children in a contaminated city and its levels are also associated with passive smoking.

## Competing interests

The authors declare that they have no competing interests.

## Authors' contributions

BLS carried out the design of the study and the acquisition of data, performed the laboratory analysis, the statistical analysis and interpretation of the data and drafted and revised the manuscript. GCS, NAL and JMGM performed the laboratory analysis, the statistical analysis and interpretation of the data and drafted and revised the manuscript. ENL participated in the acquisition and analysis of data and the laboratory analysis. DRA participated in the acquisition and analysis of the data. XSRG, RMT, participated in the design and coordination of the study and helped to interpret the data and to draft the manuscript. All authors read and approved the final manuscript.

## Pre-publication history

The pre-publication history for this paper can be accessed here:

http://www.biomedcentral.com/1471-2466/14/111/prepub
